# Collectanea Medica

**Published:** 1812-07

**Authors:** 


### COLLECTANEA MEDICA,

CONSISTING OF

### ANECDOTES, FACTS, EXTRACTS, ILLUSTRATIONS, QUERIES, SUGGESTIONS, &c.

RELATING TO THE

*History or the Art of Medicine, and the Auxiliary Sciences.*

### *Biographical Sketch of the late* MAXWELL GARTHSHORE, M.D. F.R.S. *and* S.A. M.R.I. *&c.*

IMPARTIAL records of the principles and manners of distinguished characters have long been considered as the writings most beneficial to society. They often unfold those original traits, and even eccentricities, which contribute to modulate or give a bias to the characters of succeeding sons of genius. It has also been laid down as a general proposition, that the literary portraits of such persons should rather be drawn by their friends than their enemies, because good men are much better than even their friends believe them to be, and bad men much worse than even their enemies suppose them. The position will appear more defensible, if we insist on the portrait being sketched from nature, as indispensable to the fidelity of the likeness, and admit that all hu-

 man man judgment is biassed and partial. Hence, in one case the credible portion of good will be related, in another the incredible quantity of evil concealed, and society be equally benefited by the details. The character of the pre-eminently good, however, still suffers in public estimation ; the mass of mankind reluctantly admit the existence of any thing better than themselves, and the general disposition to reduce every thing to our own standard acts as a counterpoise to the ascendancy of mere moral goodness. Without, therefore, either attempting or expecting to raise the popular criterion of human character, we shall only endeavor to relate facts, or state opinions, with that simplicity and respect for truth which become the subject.

Dr. Garthshore was born near Kirkcudbright, on November 9, 1731 (new style), and died in St. Martin's Lane, London, March 1, 1812. His father was a clergyman of the church of Scotland; he published some detached sermons, which, although perhaps inferior in literary merit to those preached in Scotland at the end of the 18th, are certainly much superior to any contemporary one delivered at the close of the 17th, century. That he was a man of learning, talents, and profound piety, cannot be doubted; and the excellent moral principles which he early inculcated in his son were remembered by him with filial gratitude to his last moments. The salutary effects indeed of an enlightened piety, in both father and mother were happily displayed in the whole life of their son. The name of Garthshore, it is believed, is now extinct, although it was once known and distinguished in the North. A few years ago the secretary to the Society of Antiquaries (Mr. Brand) read a curious grant of an estate by an Earl of Buchan to a Mr. Garthshore of Garthshore, a parent of the subject of this memoir. Some of the particulars are recorded among the transactions of learned societies in the Philosophical Magazine.

The education of Dr. G. was marked by that practical solidity which characterises the Scottish schools. His knowledge of the classic and modern languages was general and comprehensive; and he could read, write, or even speak, them with considerable ease and perspicuity. His medical studies were directed by the once celebrated Cullen; and his reputation for skill, assiduity, and humanity, had extended over several of the northern counties of England prior to 1764, when his inaugural dissertation* "De Papaveris Usu, tam

* Several German writers on opium have quoted this thesis with approbation, and it may be observed that it appeared long before the Brunonian theory had any existence. The salutary effects of laudanum tam noxia quam salutari, in Parturientibus ac Puerperis," procured him the Edinburgh academic honors of a doctor of physic. This dissertation is dedicated with much gentlemanly good humor and unfeigned respect to his early and unchangeable friend the late Sir George Baker, and does no discredit to the author's head or heart. All his friendships, indeed, being disinterested, were permanent, and he had the happiness of discovering near the same period the talents, and patronising the merit, of the late Dr. Pulteney, who at his instance obtained an Edinburgh degree without having regularly attended any college lectures. It would, we fear, be a vain effort to look for such exalted principles of friendship among any three living physicians, as distinguished, during their respective lives, Sir George Baker, Dr. Pulteney, and Dr. Garthshore. All of them were fortunate practitioners: but Dr. Pulteney realised a large fortune in a country town; Dr. Garthshore distributed one to the poor of the metropolis: the former devoted his leisure hours to botanical researches; the latter to patronise science and support scientific or humane institutions.

After Dr. G. was established as a practising physician in London (about 1764), and a licentiate of the Royal College of Physicians, he became a Fellow of the Royal Society and of the Society of Antiquaries, the meetings of which he regularly attended, and was not perhaps absent one night, except the two meetings previous to his death, during the last forty years. His insatiable desire of knowledge, and his intellectual energies, continued to the last; he read every new publication of merit, either on medicine, chemistry, general science, or divinity; and he was familiar with all the new discoveries in natural philosophy, anatomy, or the practice of medicine. Many of the modern novelties, indeed, he could trace to their original authors; the curious and obscure work of Wallace on the numbers of mankind, which perhaps gave birth to the famous Essay on the principle of Population, he had examined when writing on "Numerous Births;" and the internal use of cantharides, which has been brought forward as an original discovery in the 19th century, he well knew was ably discussed by Dr. Greenfield, a physician of the 17th, and strongly recommended by him in gleet, leucorrhœa, &c. The revived doctrines respecting the functions of the liver, use of digitalis, sea-bathing, &c. were equally known to him from their original laudanum in certain stages of child-bed are now universally admitted. Professor J. C. Starke, of Jena, in 1781, noticed Dr. G.'s opinions of the use of opium, as a respectable authority, in his medical dissertation on this subject.

sources.

sources. Hence, he was at all times able and willing to avail himself of every thing plausible that was offered as likely to mitigate the sum of human suffering, or improve the healing art. Yet, notwithstanding his indefatigable and extensive reading, his vast field of observation, his natural promptness and extraordinary accuracy, joined with habits of acute correct reasoning and judicious reflection, he published but comparatively few works on professional subjects. His experience and practice were very considerable, as the relief of the poor deeply engaged his feelings, and drew from him those interesting accounts of "Two Cases of retroverted Uterus," addressed to Dr. Hunter, and published in the 5th volume of "Medical Observations and Inquiries," 1776; a "Case of difficult Deglutition, occasioned by an Ulcer in the Œsophagus;" and of a "Species of Erysipelas followed by Gangrene, which appeared in Infants at the British Lying-in Hospital." The last two cases were described in the first and second volumes of "Medical Communications," published by Johnson, in 1784 and 1796. His "Observations on extra-uterine Cases, and on Ruptures of the Tubes and Uterus," which appeared in the Medical Journal, vol. viii. for 1787; and also a paper read to the Royal Society, and published in its Transactions for that year, on "Numerous Births," demonstrate his intimate acquaintance with both nature and art, his applicable knowledge of all that had occurred in his own practice, or that of his friends or correspondents in Paris, Montpellier, Italy, Vienna, Germany, and St. Petersburgh. But his time was too much occupied in professional practice, in conscientiously discharging the duties of a *médecin expectant,* to waste it in transcribing and polishing his journals for the press: the recovery of his patient engrossed his whole care and attention, without any regard to literary fame; the production of a flowery narrative, the rounding of a period for some new publication, or multiplied editions of a treatise on a *fashionable* disease (for among the doctors fashion is as paramount as with the milliners and mantua-makers) in order to afford opportunities of repeatedly advertising his name, were considerations very far beneath him. In this age of speculation and adventure, such things, however, are too common; but the interests of society require that they should be stigmatised, and that the memory of those who virtuously spurned them should be endeared. The sons of Britain sigh under the dire effects of modern charlatanism; and, should the manufacture of physic doctors continue to flourish as it has done of late, all the apprehensions of Mr. Malthus and his votaries, respecting overgrown population, must vanish, and sink under the manslaying operations of medical medical artifice. The sword has cut off its thousands, the doctors their tens of thousands.

But the history of medical practice during the last fifty years is too intimately allied with the venerable life of our octogenarian, and too honorable to his talents as a philosopher, and his probity as a man, to be wholly overlooked in this brief sketch. Dr. Garthshore lived to see the rise and fall of the spasmodic theory and of antispasmodics, that of the antiphlogistic regimen, phlebotomy, diffusible stimuli, drastic purges, gases, digitalis, and even stramonium! The curative powers of all those sovereign remedies, and many more, which owed their existence to the sanguine imaginations of speculative enthusiasts, have sprung up, flourished for an hour, and sunk to lasting oblivion. While popular attention, however, was occupied with these phantoms, the true indications of nature were entirely neglected, and all our knowledge of her œconomy remained as stationary as if the chemist's laboratory or the apothecary's shop had been the real seat of every disease. Hence the great merit of Dr. G.'s practice, which was peculiar, original, and efficacious, far beyond vulgar belief. Well knowing the impotency of drugs, and the imperfection of human knowledge, he studied nature, identified himself with his patient in family digressions or friendly inquiries, discovered his latent sympathies and idiosyncrasies, ensured his perfect confidence by his suavity and tender attention, engaged his feelings, relieved his mind, and restored him rapidly to health, strength, and improved moral character! In doing this, however, he was far from neglecting any other means which could possibly contribute to the relief of his patient; but he never considered them as alone sufficient, without his own personal care and gratifying attention. The native benevolence of his heart, indeed, impelled him to use every possible resource for the relief of the suffering, and his long experience added to his means, and even to his zeal in applying those means. That he was eminently successful will, we believe, be readily admitted by all disinterested persons capable of judging. Considered as an obstetrician, his talents and success peer above all competition; and he formed a distinguished exception to the truth of the general observation, that all accoucheurs are superstitious. Were we called upon to write an epitaph on his tomb, we should say, "Here lie the mortal remains of a most charitable and benevolent man, who combined the scientific talents of the philosopher with the exalted purity of the Christian, and cured more patients and killed fewer than any other physician of his age and country." Being a most a most conscientious man, he was a diffident practitioner, and he always administered powerful drugs or known poisons to his patients with a caution and personal care which, for the sake of humanity, we wish were more generally adopted or universally imitated.

With an active, vigorous, and independent mind, it was not to be expected that he could entertain respect for those corporate bodies which have no internal stimulus but selfishness. He was decidedly hostile to those interested combinations of men ycleped "Medical Societies;" all of which were anxious to insert his respectable name in the lists of their members; but they were often obliged to make very humble apologies for the liberty, and never received from him any sanction in their sordid projects of extending individual practice. Yet, whatever could by any honorable means contribute to the advancement of science, or the improvement of the healing art, invariably and promptly received his active support. It was not in his nature, indeed, to refuse assistance to any measure honestly designed for public utility; but his perception was too quick, and his judgment too correct, to be deluded by any false pretences of visionary good.

As a liberal patron of science, his memory will long be cherished by its votaries: his attention to the Royal Society, and his extensive support of the Royal Institution, evinced his zeal in the general cause of knowledge; while his Monday-evening conversations, conducted on the most laudable principles, contributed to diffuse useful truths, scrutinize medical or scientific facts, bring philosophers and men of taste into habits of mutual communication, and destroy all those petty jealousies, narrow-minded prejudices, and envious broodings, which too often disgrace the professional character. For these and many other good services to genuine philosophy, every friend of science will heave a grateful sigh to his memory, and at the same time breathe a prayer for the protracted life of his steady friend the worthy President of the Royal Society. Both kept open houses for the learned of all nations, both devoted their lives and fortunes to the melioration of their species, and both have obtained the esteem of great and good men. The patronage of Sir Joseph Banks, indeed, has been much more extensive and more glorious, but could not be more zealous, than that of Dr. G.; and the latter often regretted the inadeqnacy of his means, which prevented him from being more directly useful to the general progress of philosophy. Even his age did not abate this propensity; but his zeal for science never led him to forget the sufferings of the indigent or unfortunate, and and during the latter part of his life he distributed in private charities, and subscribed to humane institutions, above a thousand a-year, besides his gratuitous prescriptions and advice to the poor. Nor was this the consequence of instantaneous feeling, the mere impulse of the moment, but a fixed principle of action, the discharge of a Christian duty; and it is now known that at an early period of his life, and when his fortune was very limited, he gave in one charitable donation to the full amount of a year's income! To practise rather than to speak of beneficence was his uniform principle, and his generous heart and hand always gave to the utmost extent that his cooler judgment could approve. His habits of life were the most exemplary and regular. At four o'clock every morning he commenced his studies in his library till a few days before his death: this was the time allotted to write his Diary, which he kept near sixty years. His temperance was scientific, his œconomy the result of rational reflection* and extensive charities to the distressed; and perhaps no physician in London ever contributed so liberally, and with so much extreme delicacy, to the necessities of the widows, orphans, or indigent relations, of his professional brethren. So numerous, indeed, was this class of pensioners, and so liberal was his assistance to them, that, even if the disappointment of dishonest expectations should absorb the noble emotions of gratitude, the painful feelings of their loss must draw forth involuntary tributes to his memory during the remainder of their lives. Viewed as a philanthropist, as a man of talents, whose genuine simplicity and graceful ease made him equally the friend of the poor and of the great, his character rises the more it is investigated; and, if we examine his domestic piety, his clear and rational conviction of the great truths of Christianity, we shall perhaps long look for a greater and a better man in any age or country. To youth he was a most able and propitious friend: his meekness rendered him lenient to their foibles, his sagacity anticipated their wants, while his purity checked the recurrence of evil propensities. Had he possessed less virtue, he would have appeared a greater man; had he been more ambitious, vain, or reserved, his reputation might have been

* It is possible that there may be some persons ignorant enough to censure him in this respect; but, if there be, it can only prove their incapability of discriminating what should be the conduct of a philosopher and a Christian from that of a common *parvenu*. Such, indeed, is the progress of extravagant luxury, that some vulgar minds begin to think it a necessary duty!

higher, higher, but his merits and talents less. Like all conscientious men, he was extremely diffident; in many cases, even with all his knowledge and experience, he hesitated where a forward and thoughtless youth, or an impudent and ignorant quack, decided with all the dogmatism of unprincipled impostors. The horrid sentiment of 'Kill or cure,' never once found a place in his mind, as he could find in those cases no such satisfactory evidence of cures as of deaths. He was, indeed, fully sensible of the degraded state of medical practice in the metropolis, as well as the imperfect knowledge of medicine, and he zealously assisted Dr. Harrison in all his attempts in the sacred cause of medical reform. Even the case of a man dying in an English hospital by the bite of a snake, he justly considered as a stigma which every honorable practitioner should labour to remove, by qualifying himself to prevent the recurrence of a similar catastrophe. In this he was consistent, as well as in the whole tenor of his life. His last moments also were worthy of himself; and in the judicious distribution of the very moderate fortune which he left, he did not rob his relations and friends to gratify any posthumous vanity by popular bequests.

A respectable Magazine, in a memoir of Dr. G., otherwise authentic and correct, states, either in consequence of a typographical error or wilful misrepresentation, that the doctor died worth 55,000*l.* Every person who wishes to know the truth may learn, by applying at the proper office, that the whole amount of his property was only about 35,000*l.* (*Phil. Mag.*)

---

The right of Licentiates of the Royal College of Physicians to be regarded as members of the college, and to claim the privilege of the fellows in being exempt from bearing arms, having been disputed, and in one or two instances, of no remote occurrence, a fine having been paid by a licentiate of said college for finding a substitute for the militia, we are induced to give the following authentic documents on the subject from Dr. Goodall's account of the college (ed. 1684).

"In the 10th year of king Henry VIII. the physicians of London and within seven miles of the same, upon many important reasons mentioned in their royal patent, were made a body corporate, and endowed with many privileges, which in the 14th and 15th of his reign were confirmed by act of parliament.

In the 32d of the same king's reign, several additional privileges were granted them by a second act of parliament, by which they were discharged from keeping any watch and and ward, or being chosen to any office in London or the suburbs thereof, and were thereby enabled to practise surgery as well as physic in the said city, &c. By which clause, they were entituled to the privilege granted the surgeons of being discharged from bearing arms, &c. by the 5 Hen. VIII. 6.

In the 1 Q. M. 9, their charter was a second time confirmed by act of parliament, and additional privileges granted to them; which privileges, with a freedom from finding arms, were continued to them without interruption till 1588; and, it being then a time of most imminent and publick danger, the lord mayor of London and court of aldermen charged the college with arms, whereupon they applied themselves to Queen Elizabeth and her council: upon which Secretary Walsingham wrote a letter to the lord mayor and aldermen of London, that they should no more trouble the college, but permit them to live quietly and free from that charge. After this, they met with no farther trouble or molestation till the reign of King James, at which time, the college being charged with arms, Sir William Paddy pleaded their privilege before Sir Thomas Middleton, lord mayor, and a full court of aldermen, and Sir Henry Montague, recorder." P. 282-3.

The following is an account of the proceedings on that occasion:

"In the 12th year of King James his reign, some of the members of the college being required to find arms, the college appointed two of their fellows, viz. Sir William Paddy and Dr. Lister, to solicit their cause (with the recorder of London, the lord mayor, and court of aldermen) in the behalf of the fellows, candidates, and licentiates, for immunity from the charge of service for men or armour. Whereupon Sir William Paddy, accompanied with Dr. Lister, before Sir Thomas Middleton, knt. then lord mayor, and a full court of aldermen, upon the 4th of October, 1614, after a short preamble made (that is to say, that the fellows of the College of Physicians of London became suitors unto the lord mayor and that honorable court, that it would please them to take into their considerations the privileges granted unto the college by acts of parliament, whereby they were, as heretofore they have been, exempted from the charge of service) proceeded to the reasons following.

First, applying his speech to Sir Henry Mountague, recorder for the city, he desired him that he would indifferently peruse the words in the preamble of the act of parliament recited thus, *In consideration, &c.* Herein may it please this honorable court, not onely all articles, graunts, and other things contained in the letters patent, but also for enlargement enlargement of further articles for the said college, are to be interpreted available to the said college, in as large and ample manner as may be taken, thought, and construed, by the same. 14 Hen. VIII.

Then may it please you to observe, that in the 32d of H. VIII. they, and every of them of the said body corporate or fellowship, and their successours, shall at all time and times after the making of the said act, be discharged to keep *any* watch or ward in the said city of London, or the suburbs of the same. And here the said Sir William requested them to note the word (*any*) which in true weight of construction was to be extended, as if that clause had been in more words expressed.

Then he farther urged that for the chirurgeons, where, in the first entrance of the act, it was thought expedient by the wisdom of the land to provide for men expert in the science of physick and chirurgery.

And therefore, when it followeth that in this act of parliament the chirurgeons by express word are exempt from the bearing of armour, it may truly infer that physicians are exempted (as before) from any watch or ward; as also physicians here recited in the preamble should receive a greater, or at least the same, immunity, especially since physicians are by their science chirurgeons without further examination, and approbation to be had from the bishop of London, whereunto many chirurgeons are subject.

Then a grave and reverend knight, an alderman of the bench, replied, that he took the words in the act of parliament for the chirurgeons (viz. bearing of arms) were to free their persons, and not to exempt them from the charge of the service.

Whereunto Sir William Paddy answered, under his favor, and the judgment of the bench, and Mr. Recorder, that the difference between bearing and wearing of armour was such, that the very *etymon* of the word *bearing*, as in many other cases comprehended both, and therefore should give immunity from both.

And therewithall Sir William Paddy added the reason, that by the wisdom of the land it must needs be intended physicians of the college should be exempted from this and other like services, for that, in the time of all outward war or domestick, they or some of them do attend the armies in person; whereof he there exhibited a catalogue of divers he had from the register. And now may it please you my lord mayor and this honorable court, we address ourselves onely to you, under whose government we are seated, and with love we seek from you favorable construction for just

 relief; relief; which, as in your worth you have always afforded to all, so do we assure ourselves you will dispense unto us, who live best by your love, and will ever be ready to do you service.

Then Mr. Recorder, perusing every branch of the statutes recited, and the reasons urged, and opening every part thereof at large, did gravely and judicially conclude, that the acts of parliament did merely intend to give to the college as much immunity as in any sort to the chirurgeons.

Whereupon the court desired to have a true catalogue of the fellowes, candidates, and licentiates, in number then forty and one, which Sir William Paddy and Dr. Lister, from the register, did immediately deliver up unto them; which catalogue the court then (upon this reason) required, lest others not of the college should delude them, and so claim privilege.

Hereupon was ordered a dispensation for the college from bearing of arms, and immediately after a precept was awarded by the lord mayor and court to commit all other physicians or chirurgeons refusing to bear or find arms, who were not by the college allowed, or chirurgeons licensed according to form." P. 378–380.

*Extracts from the College Annals.*

" *Comit. Maj. Ord. Dec.* 7, 1711.—The attorney-general, Sir Edward Northey, gives his opinion, that neither the college, nor any member thereof, are obliged to provide arms.

" *Comitiis Majoribus Ordinariis. Sept.* 30, 1730.—The president acquainted the college he had leave from his grace the Duke of Newcastle, lord-lieutenant of the county of Middlesex, to assure the college, that for the future no member of the college should be molested on account of the militia of Middlesex."

---

*Elastic Gum. Caoutchoc.*

The tree which produces this singular substance, the history of which was inquired after in a former number, by our correspondent A. B. is of the class *monoecia*, ord. *monadelphia*. It is the SIPHONIA CAHUCHU* of Linnæus, (Spec. Plant. Willd. 4. p. 567.) growing in the Brazils and Guiana. We are

---

* Synom. Jatropha elastica. Linn. Suppl. 422.—Hevea guianensis. Aublet, Guian. 2. p. 871. pl. 335.—Poa seringa. Act. Paris, 1751. pl. 20, figura mala.

In order to give our correspondent farther information on the natural and botanical history of this species of *Siphonia*, we insert the following account from Mr. Martyr's Dictionary.

"The are indebted to the 3d volume of Mr. Wood's Zoography for the following circumstances respecting it.

"The *Siphonia Cahuchu* is the only known species of the genus, and has been mentioned by several naturalists. Amongst others, Aublet has noticed its fruit, and the milky resinous juice which exudes from its trunk; but Richard, a French botanist, was the first who described the flower, no one before his time having paid any particular attention to that part of the plant. Each flower-stalk bears a great number of male flowers, with one solitary female at the top. Neither the

---

"The tree yielding that resin which is now commonly known under the name of elastic gum, is supposed to be of the genus *jatropha*, but Schreber has placed it a new genus, *siphonia*. It is a native of Guiana, of Quito, and Brazil, particularly in Para, where it is called massaradub. The Indians, by an incision in the bark, extract a viscid white substance, like that which issues from the fig-tree (there are several other trees which yield a similar juice, as the Ficus indica, Cecropia peltata, &c.); they receive it into earthen molds, to make rings, bracelets, syringes, &c. &c. Abbé Rochon says, that the inhabitants of Madagascar also made flambeaux of it, which burn without wicks, and afford them a very good light when they go out to fish in the night-time; and that in a state of solution it is very proper for coating over silk, to render it impervious to air or water. It dissolves in ether and linseed oil, and in nut oil digested gently in a sand bath; there are also other fat and oily substances which affect it very sensibly. The Chinese have been long acquainted with the art of dissolving it, and of giving it various colors. Abbé Rochon describes the tree which yields the elastic gum in Madagascar as twenty feet high, the leaves eight inches long and two inches broad, the fruit resembling a round fig and full of small seeds. We may conclude, therefore, that this is the *Ficus Indica;* certainly not a jatropha. The coagulated juice, however, is of the same nature.

"The first account which we have of the caoutchouc, as it is called by the Indians to the S. E. of Quito, is in M. de la Condamine's relation of the river of Amazons, in 1745. The tree which yields the elastic gum grows along the banks of this river, and is very common in the province of Emeralds to the N. of Quito, where it is called *Hheve,* from whence Aublet has taken his generic name. The foliage is not unlike that of the Cassava. The fruit is triangular, inclosing three seeds. These seeds, or kernels, peeled and boiled in water, yield a thick oil, which the Indians use as butter with their food. M. Fresnau likewise, about 1746, discovered the same tree at Cayenne.

"Whatever is made with this substance, must be done on the spot, because it thickens and dries very fast. Warm water will soften and render it very supple; *the heat of the sun does not make any impression* on it, but it is sensible to the least degree of frost."

one one nor the other have any corolla; but there is a bell or cup shaped calyx in the room of it, with five teeth. There are five stamens to each male flower, of which the filaments are united into a little cylindrical column, much shorter than the calyx, carrying thin oval anthers a little below the summit of the column. The female flower has no style, but merely a superior germ of a globular and conical figure, upon which we find three flat stigmas. The fruit is a capsule consisting of three ligneous husks, each of which inclose two or three white seeds of a pleasant flavor, wrapped up in a thin and brittle coat.

"This tree is described as very straight and tall. According to Aublet, it grows to the height of fifty or sixty feet. The trunk measures two feet and a half at the base, and the branches, which shoot out from the top, grow in all directions. The leaves are oval, thick, and tough; growing three together upon the same leaf-stalk, and having both surfaces glazed, but of a different color, the superior surface being green, while the inferior is cinereous. There is a variety of this tree, consisting chiefly in the leaves being smaller, of a more pointed oval, and very thin.

"The syringe tree, which has long been celebrated for its elastic resin, or caoutchouc, has been described by M. de la Condamine in a memoir, accompanied with a figure of the leaves and fruit, in the *Recueil de l'Academie des Sciences* for the year 1751, from which we learn that this academician found a considerable number of these trees in the forests of South America, to the north of Quito, where the natives of the country have given them the names of *heve*. When the tree is wounded, there exudes from the incision a white liquor, like milk, which gradually hardens in the air; of this the inhabitants make their flambeaux, which are about an inch and a half in diameter and two feet long. These flambeaux burn very well without any wick, and give a clear bright light; they exhale, during their consumption, a particular, but not disagreeable, smell; and one of them, we are assured, will continue to burn for twelve hours. In the province of Quito they prepare their linen and canvass with this resin, so as to make it answer all the purposes of our oil-cloth.

"Along the banks of the river Amazon, where this tree is not uncommon, the natives form the resin into rude figures of fruit, birds, and objects of different kinds. They likewise make their boots of it, which, being completely waterproof, are highly serviceable to the inhabitants of a country where the rains are heavy, and the plains intersected with rivulets. In the interior of the American continent the inhabitants habitants mould the resin into bottles, to the necks of which they affix wooden pipes, and thus construct complete syringes; from which the Portuguese of the colony of Pava have called the tree *Poa de xeringa.* The wood of this singular vegetable production might be wrought into small masts, as it is both light and straight.

"We learn from Bomare, that M. de la Borde, who travelled by order of the French government, in 1772, into the interior of Guiana, discovered this tree growing on the banks of lakes and rivers. They are not easily distinguished in the woods, from their tufted branches being intermixed with the surrounding foliage; but nevertheless they may be detected by the quantity of young plants which are produced from the seeds, and which, after having increased to a certain size, are overshadowed by the forests, and perish for want of room and air.

"The resinous juice of this tree, according to M. de la Borde, flows at all times of the year, but the rainy season is the most favorable for collecting it; and this is the time that Indians choose for the purpose. They begin their operations by washing the foot of the tree to the height of seven or eight feet; then they bind the trunk below the place where they begin to wash, with a cord about the size of the little finger. This serves to support a layer of moist earth, in which they contrive a trench, and place in it a palm leaf as a gutter, the end being immediately over the calabash resting on the ground. Having proceeded thus far, and disposed all things in order, they cut several gashes in the trunk of the tree, to the height of three feet, and the juice trickling from the wounds into the trench is carried along the palm leaf, and falls into the calabach prepared for its reception. When the tree ceases to furnish the juice, the Indians proceed to a preparation which they are careful not to disclose; this is the manner in which they pour it upon the clay-moulds, made on purpose, where it acquires that consistence and form which render it applicable to the different purposes for which we require it in Europe.

"In making a bottle or other vessel, they are said to line the mould with the prepared juice while it is yet liquid, and expose it to a thick smoke till the lining becomes of a yellow color; upon this they put a second layer, which is treated in the same manner; and they judge from experience when their vessel is of a proper thickness. When the resin is dry and hard, they pick out the clay and fill the elastic bottle with water, in order to wash out the pieces of the mould that may chance to remain in the cavity. In this state the substance, substance, which is very flexible and almost insoluble, is called elastic gum.

"Luke-warm water, or a heat of eighty or a hundred degrees, softens this substance, and softens it more or less according to its thickness; but never renders it fit to be moulded anew. This part of the process it would be highly gratifying to discover; and we should be tempted, were it in our power, to rob the Indians of their secret, that so valuable and singular a commodity might be moulded by Europeans.

"Many different menstrua have been tried to dissolve this singular substance, but none seems to have succeeded completely except ether. This fluid dissolves it without any other heat than that of the atmosphere, and produces a transparent and amber-colored solution. To succeed, however, in this operation, the ether must be of the very best quality. Spirit of turpentine seems to be the next best menstruum for this purpose, since, with the assistance of heat, it may be made to dissolve the elastic resin, provided only a small quantity, and that cut very thin, is exposed to the spirit at a time."

